# Timing of population peaks of Norway lemming in relation to atmospheric pressure: A hypothesis to explain the spatial synchrony

**DOI:** 10.1038/srep27225

**Published:** 2016-06-01

**Authors:** Vidar Selås

**Affiliations:** 1 Department of Ecology and Natural Resource Management, Norwegian University of Life Sciences, P.O. Box 5003, N-1432 Ås, Norway

## Abstract

Herbivore cycles are often synchronized over larger areas than what could be explained by dispersal. In Norway, the 3–4 year lemming cycle usually show no more than a one-year time lag between different regions, despite distances of up to 1000 km. If important food plants are forced to reallocate defensive proteins in years with high seed production, spatially synchronized herbivore outbreaks may be due to climate-synchronized peaks in flowering. Because lemming peaks are expected to occur one year after a flowering peak, and the formation of flower buds is induced in the year before flowering, a two-year time lag between flower-inducing climate events and lemming peaks is predicted. At Hardangervidda, South Norway, the probability that a year was a population peak year of lemming during 1920–2014 increased with increasing midsummer atmospheric pressure two years earlier, even when the number of years since the previous peak was accounted for.

The pronounced multiannual population cycles of small rodents and other herbivores in the Northern Hemisphere have commonly been attributed to predation by specialist predators, possibly in combination with overgrazing in the peak phase. However, for Norway lemming (*Lemmus lemmus*, hereafter lemming), which inhabits alpine and arctic areas, the famous 3–4 year cycles are found also in areas where the specialist predators (small mustelids) are absent^1^. There is no doubt that predators may depress rodent numbers in the decline phase of the cycle^2^, or that small rodents in peak years may reduce the biomass of food plants^3^, but these patterns would be expected also if rodent cycles are generated by fluctuations in food quality, caused by factors other than herbivory^4^. Plant chemistry is, however, a complex topic, and traditionally analyses of plant nutrients, crude protein, fibre or phenolic content^5^ may not be appropriate to document a plant’s nutritional value^6^.

Because plant tissue in general has low digestibility and protein content, the protein digestibility per time unit is crucial for herbivores^7^. Protein availability is particularly important for reproduction, and thus for the population growth of small rodents^8^,^9^. According to the plant stress hypothesis^7^, any stress factor that requires increased metabolic activity in a plant will force the plant to reallocate complex plant proteins, stored as feeding deterrents^10^, to transportable and easily digestive proteins. One stress factor suggested to have this effect is high seed production^11^, termed masting or quasi-masting if more or less synchronized within a plant population. If, as a consequence of masting, the ratio of digestive to defensive proteins rises above the critical threshold for herbivore reproduction in a plant population, also less preferred plants may become suitable as food, by serving as supplemental energy sources. As a parallel, reindeer (*Rangifer tarandus*) fed solely on lichens lose body mass, but maintain it if they get a limited supplement of more N-rich feed^12^.

Also predation^2^ and unfavourable weather conditions^13^,^14^ affect rodent populations. However, if the plant stress hypothesis is correct, these factors should affect only the shape and the amplitude, and not the timing or the period, of population cycles. One striking feature of rodent cycles is that they are often synchronized over much larger areas than what could be explained by dispersal^15^,^16^. In Norway, population peaks of lemming usually show no more than a one-year time lag between different regions, despite distances of up to 1000 km^17^. They are also commonly synchronised with population peaks of other small rodents^18^. During 1955–2005, 13 of 15 lemming peaks at Hardangervidda, South Norway^13^,^19^,^20^,^21^, overlapped in time with peaks of an allopatric bank vole (*Myodes glareolus*) population in a lowland forest area in the southernmost part of the country^11^,^22^.

It is well established that populations of *Myodes*-voles reach their peak one year after a peak in seed production of dwarf shrubs such as bilberry (*Vaccinium myrtillus*)^22^,^23^,^24^. Lemmings feed mainly on other plants than dwarf shrubs, such as mosses, grasses and sedges^25^, but also within these plant groups some species show large inter-annual variations in sexual reproduction^26^,^27^,^28^,^29^. If lemming numbers are kept at a low level because of low food quality, a significant population increase would be expected even if the protein availability increases above the critical threshold for reproduction only in one or a few common plant species. For sedges of the *Carex bigelowii* complex, which are commonly grazed by lemmings^25^, the flowering frequency is highest prior to the peak phase of the lemming cycle^30^, and the lemming density is negatively related to the level of trypsin inhibitors in these plants^31^.

Regardless of the causal link, spatial synchrony in both plant reproduction^26^,^32^ and rodent peaks indicates that sexual reproduction in plants is induced by some large-scale environmental factors. Temperature and day-length one year before flowering are regarded as important factors for flower induction in dwarf shrubs, grasses and sedges grazed by small rodents^26^,^33^,^34^,^35^. The question is whether the pattern in temperature fluctuations is sufficiently consistent between different regions of Norway to explain the synchrony of rodent cycles. However, in northern areas, temperatures are strongly connected to atmospheric pressure, which usually shows synchronous variation over larger areas than temperature measurements^36^, and which influences also other environmental factors that may affect plant flowering, such as light quantity and quality^37^.

Here, I propose the hypothesis that atmospheric pressure represents some signals that trigger flowering in important lemming food plants in Norway, and thus act as a spatially synchronizing factor for the lemming cycle. Because lemming peaks are assumed to occur one year after flowering peaks of the food plants in question, and the formation of flower buds is induced in the year before flowering^34^, a two-year time lag between atmospheric pressure and lemming peaks is predicted. I test this prediction by comparing the pattern of reported lemming peaks, for which there are much longer time series than for any records on plant reproduction, with annual variations in atmospheric pressure.

## Results

**Figure 1:**
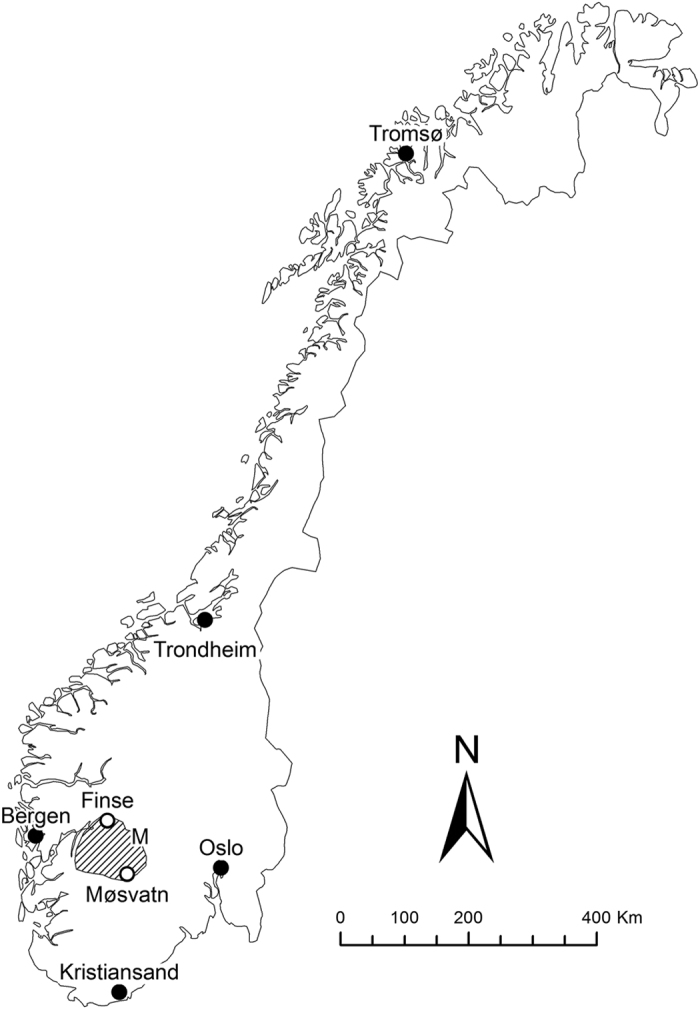
Map of Norway showing the location of Hardangervidda (hatched area), the snap trapping stations Finse and Møsvatn, the closest meteorological station (M) and the cities with atmospheric pressure measurements used in the analyses. The map was generated using ESRI’s ArcGIS Desktop ArcMap 10.3.1 software (http://www.esri.com/).

**Figure 2:**
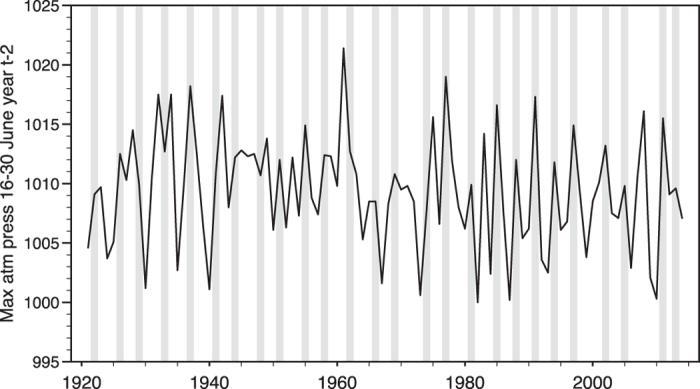
Lemming peak years (bars) at Hardangervidda, South Norway, compared with maximum atmospheric pressure 16–30 June two years earlier. For cases with two consecutive years with high lemming numbers (see text), only the first one is shown in the figure.

The most complete record of lemming population peaks in Norway is from the 8000 km^2^ mountain plateau Hardangervidda in Telemark, Buskerud and Hordaland counties, South Norway (60°N, 7–8°E, general altitude 1100–1200 m; Fig. 1). During 1921–2014, there were 26 lemming peaks (Fig. 2), with a significant regular periodicity of 3.6 years (Fisher’s Kappa = 15.15, *P* < 0.001). The probability of a year being a lemming peak year increased with the number of years elapsed since the previous peak (χ^2^ = 17.10, *P* < 0.001) and with atmospheric pressure 16–30 June two years earlier (mean pressure: χ^2^ = 5.94, *P* = 0.015; maximum pressure: χ^2^ = 14.48, *P* < 0.001; Fig. 2). It was not significantly related to atmospheric pressure in July–September, or to the mean temperature in any of the summer months two years earlier.

The best multiple model to explain the probability of lemming peaks included number of years elapsed since the previous peak, maximum atmospheric pressure in late June two years earlier, and mean July temperature two years earlier, the latter with negative sign (Table 1). The second best model included only number of years since previous peak and maximum atmospheric pressure, and the third best number of years, mean atmospheric pressure and mean July temperature (Table 1). The results were essentially the same if two minor peaks, 1985 and 1997, were omitted, except that the relationship with mean temperature in July in model 3 was not longer significant (*P* = 0.074).

**Figure 3:**
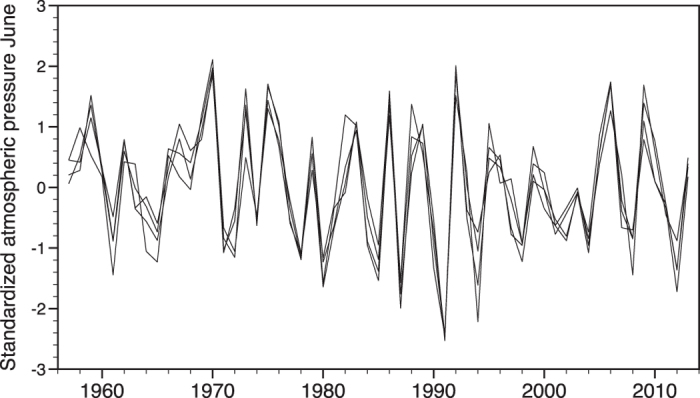
Standardized mean atmospheric pressure in June 1957–2013 at four meteorological stations (Kristiansand, Bergen, Oslo, Trondheim) surrounding Hardangervidda, South Norway.

For the shorter period 1957–2013, from which data on atmospheric pressure are available from several meteorological stations, there was a highly significant positive correlation between mean atmospheric pressure in June from the four Norwegian cities Kristiansand, Bergen, Oslo and Trondheim (Table 2 and Fig. 3). The mean atmospheric pressure in each of these cities was significantly correlated even with the atmospheric pressure in the more distant city Tromsø in North Norway (Table 2). In spectral density analyses, there was a significant periodicity of 3.3–3.4 year in mean atmospheric pressure in June in Oslo, both for the period 1957–2013 (Fisher’s Kappa = 10.25, *P* < 0.001), and for the entire period 1885–2013 (Kappa = 7.54, *P* = 0.023).

## Discussion

Atmospheric pressure in late June, i.e. in midsummer, contributed significantly to explaining the timing of lemming peaks with the expected time lag of two years, even when the number of years elapsed since the previous peak was accounted for. The fact that sexual reproduction is costly for plants^29^,^38^ makes it likely that the lemming peaks reflect improved performance due to a trade-off between reproduction and defence in food plants. Midsummer conditions being important for flower induction of plant species grazed by small rodents is in accordance with recent studies on bilberry^35^, where high temperatures in June induced formation of flower buds. For *Carex bigelowii*, high temperatures somewhat later, in July, appeared to be important for flower bud induction in Swedish Lapland^28^. This widespread sedge is common at hummocks and ridges, and will thus be among the first lemming food plants to start growing after snowmelt in spring.

An impact of environmental factors on flower bud formation in late June requires that the alpine plants in question are not covered by snow at that time. Information about snow cover at or close to Hardangervidda is available from 1957 onwards, both from the Norwegian Meteorological Institute and from the Norwegian Water Resources and Energy Directorate. Only for two of the 15 years that occurred two years prior to a lemming peak, there was more than 50% snow cover at meteorological stations situated above 1000 m elevation at the date of the highest atmospheric pressure in late June. The two exceptions were 1989 and 2000. In years with a late thaw, the time of flower induction may be postponed. For instance, in 1989, there was a very high atmospheric pressure 3–4 July, when the snow cover was reduced to less than 50%.

Although plants may respond to variations in atmospheric pressure as such^39^, it seems unlikely that this force is strong enough to have a significant impact on plant reproduction. Temperatures appear to be the favourite candidate for causing spatial synchrony of flower induction in alpine plants in Norway, but atmospheric pressure also affect several other environmental factors, such as clouds and precipitation^36^, light conditions and UV-B radiation^40^, and even cosmic ray fluxes^41^, a factor that has been hypothesized to cause decadal cycles in herbivore populations through the ionizing effect on plants^42^. Interestingly, the NAO-index, which is based on large-scale atmospheric pressure patterns, is often a better predictor of herbivore performance than temperature and precipitation indices obtained from local meteorological stations^43^,^44^. A possible explanation is that air pressure represents a “package” of weather variables that explains ecological phenomena better than what single meteorological parameters do^45^.

At Hardangevidda, some lemming peaks lasted for one year and some for two. This may depend on plant recovery, which may be affected by different environmental factors, but in particular summer temperatures^22^. At a regional scale, population cycles of lemmings and other herbivores are most pronounced in high altitude areas with low summer temperatures^22^,^46^,^47^. It is also a general pattern that the amplitude of herbivore cycles decreases in periods with high temperatures^48^,^49^,^50^. This may explain the negative relationship between July temperature and lemming peaks in the multiple models. If all years occurring one year after a population peak, regardless of whether there was still a high population level or not, were excluded from the analyses, there was no significant relationship with July temperature.

Due to different shape between lemming and vole cycles, it has been suggested that lemming cycles are caused by interactions with food plants, whereas voles are regulated by predators^51^. However, steeper increases in lemming cycles are probably caused mainly by more frequent winter reproduction in lemmings than in voles^47^. Lemming cycles also have larger amplitude variations, possibly because lemmings are more vulnerable to unfavourable snow conditions, i.e. melting and freezing^13^,^52^. The obvious bottom-up relationship between berries and *Myodes*-voles, and the fact that specialist predators are not needed to generate cycles in field vole (*Microtus agrestis*) populations^53^, do not support the view that vole cycles are predator-generated. But even if the timing and periodicity of lemming and vole cycles have largely the same origin, species-specific vulnerability to harsh physical conditions or predation may be important factors in shaping the cycle of each rodent species^47^.

Although population peaks of rodents with different diet are more often synchronous than asynchronous, there are examples of lemming peaks occurring both one year after a vole peak and in periods with no vole peak at all^18^. It is possible that perennial plants grazed by small rodents are not perfectly synchronized because of different sensitivity to climate cues acting during different stages of the flowering cycle^34^. In addition, unfavourable conditions such as late snowmelt and summer drought may disrupt the synchrony by affecting some plant species stronger than others. Nonetheless, for domestic sheep (*Ovis aries*) on mountain pastures in South Norway, where sedges and grasses are important forage, the weight gain of lambs during summer and autumn peaked in 1982, 1985, 1988, 1990, 1993, 1997, 2002 and 2004–05^54^,^55^,^56^. These peaks, which are difficult to explain by weather alone^55^, all occurred near a lemming peak year.

The 3.3–3.4 year periodicity in June atmospheric pressure in Norway corresponds to the 3.3–3.4 year period in the El Niño Southern Oscillation, which affects climate worldwide^57^. This more or less predictable fluctuation appears to be a plausible synchronizing factor for the flowering of some alpine plant species grazed by lemmings in Norway. Future research goals should be to identify the time of flower bud induction of different plant species with inter-annual variation in seed production, and to reveal their relationship with the abundance of lemming and other herbivores.

## Methods

### Identifying lemming peak years

Wildhagen^46^ identified peak years of voles (species not always given) and lemming at Hardangervidda for the period 1871–1949 based on a large number of sources, such as books, reports, articles and newspapers. It seems likely that all or most major lemming peaks were identified by this approach, but lack of information about rodent numbers for a given year or period does not exclude the possibility that there was a minor peak. From 1932 onwards, this source of error is virtually eliminated by the introduction of mandatory reporting of game population levels (including lemming) from each municipality to the central game authorities. Prior to 1932, there were three very high lemming peaks at Hardangervidda; 1922, 1926 and 1929, ccurring with the expected 3–4 year intervals^46^. I therefore included the 1920s in the analyses.

For the period 1921–1953, Østbye *et al*.^58^ regarded 1922–23, 1926, 1929–30, 1934, 1937, 1941, 1944, 1948 and 1951–52 as lemming peak years at Hardangervidda. This is in accordance with Wildhagen^46^, but the latter also considered 1933 as a peak year, which is thus included in the analyses. According to game reports from 1946–1970, there were high or relatively high lemming numbers at Hardangervidda in 1955–56, 1958–59, 1962–63, 1966 and 1969–70^19^,^20^.

The information sources cited here were used also by Angerbjörn *et al*.^17^ to identify lemming peak years for southwestern Norway, but they did not regard 1930, 1934, 1955 and 1969 as peak years (score <3 on scale 0–5). Because 1930 and 1934 were the second of two consecutive years with high or relative high lemming numbers, the inclusion/exclusion of these years have minor impact for the hypothesis of a relationship with atmospheric pressure two years prior to the population peak. When it comes to the less pronounced 1955–56 peak, the number of game reports reporting a lemming population level above average in autumn was equally high in 1955 as in 1956^19^, and I therefore included both years. Although 1970 was the peak year in most of Norway, reports of “above average” lemming numbers peaked in 1969 in western parts of Hardangervidda^20^, and this year should thus also be included.

The practice of game reports was terminated in the 1970s, but small rodents have been snap-trapped annually at Finse, situated at the northern part of Hardangervidda, from 1970 onwards^13^,^18^,^21^, and at Møsvatn, situated at the south-eastern part of Hardangervidda, from 1993 onwards^18^. Unfortunately, lemmings are to a lower extent than other rodents attracted to the baits used in snap-traps^59^. This is well illustrated by a snap-trapping study I conducted during 2002–2015 in an alpine area (Jotunheimen) situated at 1100–1200 m elevation 120 km north of Hardangervidda, with 400 trap nights each August, and raw carrot as bait. The experimental protocol was approved by the Norwegian Environment Agency, and the study was carried out in accordance with their guidelines. Even though lemmings were commonly observed in the study area in peak years, the total capture of lemmings over these 14 years was only four individuals, compared to 138 root voles (*Microtus oeconomus*), 57 field voles (*Microtus agrestis*), 337 bank voles, 116 grey-sided voles (*Myodes rufocanus*) and 133 common shrews (*Sorex araneus*). If high trapping indices of lemming are obtained only when the population level is so high that many individuals hit the traps just by running over them, then the method may fail to reveal minor or moderate peak levels.

According to the snap trapping study at Finse, there was a marked peak in the lemming population in 1970, 1974, 1977, 1981, 1988, 1991 and 1994^21^. However, there also was a small peak in the trapping index in 1985^21^, which I have included in the analyses. This year, there were high population levels of small rodents in most alpine areas in Norway^60^, and a high population level of lemming at least in northern parts of South Norway^59^. According to O. F. Steen (pers. comm.), lemmings were abundant also at Hardangervidda in early summer, but there may have been a decline before autumn, i.e. prior to the snap trapping at Finse.

From 1994 to 2014, lemmings have in general been rare in the trapping data from Finse^18^, but at Møsvatn, there was a marked peak in the trapping index in 1994, 2002, 2005, 2011 and 2013–14^18^. The peaks in 2002 and 2011 were in fact reported also from western parts of Hardangervidda^61^,^62^. If we expect a 3–4-year fluctuation pattern, a peak around 1998 and one around 2008 were apparently “lost” in both snap-trapping series. However, according to R. Borgstrøm (pers. comm.), there was a small lemming peak at western parts of Hardangervidda in 1997, when high numbers were reported from alpine areas 50–100 km farther south^63^. I therefore regarded also 1997 as a lemming peak year.

### Statistical analyses

I tested for periodicity in the lemming data, as well as in atmospheric pressure, by using the Fisher Kappa test in a spectral analysis. Relationships between lemming peaks and the explanatory variables were tested in logistic regression models, with lemming peak years and non-peak years as the response variable. The number of years elapsed since the previous peak was included as a covariate, because plants may need a minimum recovery period after each peak in seed production. This makes the tests conservative, as much of the variation in the data set may be explained by this factor.

The explanatory variables used were mean and maximum atmospheric pressure and mean temperature in June, July, August and September two years earlier. In some years, large parts of Hardangervidda are still covered by snow in early June, and therefore, also mean and maximum values from 15–30 June were used. The best multiple model was selected based on AICc-values. None of the explanatory variables entered in the final models were significantly correlated, and the only variable with a significant autocorrelation (positive at lag 6 years) was mean atmospheric pressure 16–30 June.

All weather variables used were provided by the Norwegian Meteorological Institute (http://sharki.oslo.dnmi.no/portal/page?_pageid=73,39035,73_39049&_dad=portal&_schema=PORTAL&6009_BATCHORDER_3197941). Data on atmospheric pressure from the early 1900s are available only from Ås Meteorological Station (89 m elevation), situated 20 km south of Oslo. Data are missing from a period after 1987, but a time series from Blindern Meteorological Station (94 m elevation), Oslo, from 1952–2014, shows almost identical values to that of Ås for the overlapping period. The data used in this paper are from Ås 1920–1952 and Blindern 1953–2012.

There are few high-latitude meteorological stations close to Hardangervidda with temperature data that cover the entire study period. The temperature data used are from four locations in Dagali (798–887 m elevation), Hol municipality, situated close to the north-eastern part of Hardangervidda (Fig. 1), and covering the period 1921–2014. To justify the use of atmospheric pressure data from Oslo, situated 150–200 km east of Hardangervidda, I compared them with data from three other meteorological stations, available from 1957 onwards. The stations used were situated close to the three cities Kristiansand (Oksøy, 9 m elevation), Bergen (Flesland, 48 m elevation) and Trondheim (Ørland, 10–12 m elevation), situated south, west and north of Hardangervidda, respectively (Fig. 1).

## Additional Information

**How to cite this article**: Selås, V. Timing of population peaks of Norway lemming in relation to atmospheric pressure: A hypothesis to explain the spatial synchrony. *Sci. Rep.* **6**, 27225; doi: 10.1038/srep27225 (2016).

## Figures and Tables

**Table 1:** Results from the best multiple logistic regression models with the probability of a year being a lemming peak year during 1924–2014 as response variable.

Explanatory variable	Estimate	SE	L-R χ^2^	*P*	∆AICc
Intercept	−227.60	65.61			
Number of years since previous peak	0.97	0.27	16.25	<0.001	
Maximum air pressure 16–30 June, 2 yr lag	0.23	0.07	15.31	<0.001	
Mean July temperature, 2 yr lag	−0.52	0.22	6.06	0.014	0.00
Intercept	−196.58	61.75			
Number of years since previous peak	0.89	0.25	14.84	<0.001	
Maximum air pressure 16–30 June, 2 yr lag	0.19	0.06	11.92	<0.001	3.87
Intercept	−145.69	68.39			
Number of years since previous peak	0.91	0.25	16.70	<0.001	
Mean air pressure 16–30 June, 2 yr lag	0.15	0.07	4.95	0.026	
Mean July temperature, 2 yr lag	−0.38	0.20	3.90	0.048	10.36

**Table 2:** Correlation coefficients and associated *P*-values for comparisons of mean monthly atmospheric pressure in June between five Norwegian cities for the period 1957–2013.

	Distance	Air pressure	
	(km)	*r*	*P*
Kristiansand – Bergen	292	0.96	<0.0001
Kristiansand – Oslo	252	0.96	<0.0001
Kristiansand – Trondheim	602	0.81	<0.0001
Kristiansand – Tromsø	1383	0.37	0.0041
Bergen – Oslo	306	0.95	<0.0001
Bergen – Trondheim	430	0.91	<0.0001
Bergen – Tromsø	1208	0.47	0.0002
Oslo – Trondheim	392	0.90	<0.0001
Oslo – Tromsø	1149	0.56	<0.0001
Trondheim – Tromsø	788	0.74	<0.0001

The data from each city are taken from the closest meteorological station with atmospheric pressure measurements.
